# Circulating miR‐144‐3p as a Novel Independent Biomarker Associated With Low Muscle Strength Among Older Adults

**DOI:** 10.1002/jcsm.70026

**Published:** 2025-07-29

**Authors:** Hyung Eun Shin, Chang Won Won, Gustavo Duque, Miji Kim

**Affiliations:** ^1^ Department of Health Sciences and Technology College of Medicine, Kyung Hee University Seoul Republic of Korea; ^2^ Elderly Frailty Research Center, Department of Family Medicine, College of Medicine Kyung Hee University, Kyung Hee University Medical Center Seoul Republic of Korea; ^3^ Bone, Muscle & Geroscience Group Research Institute of the McGill University Health Centre Montreal Quebec Canada

**Keywords:** components, miRNAs, muscle strength, older adults, profiling, sarcopenia

## Abstract

**Background:**

Low muscle strength, a key component of sarcopenia, is significant in the development of adverse health outcomes among older adults. MicroRNAs (miRNAs) have been implicated in mechanisms of sarcopenia; however, their specific functions in sarcopenia components, particularly low muscle strength, remain unclear. We aimed to examine distinct miRNA signatures associated with muscle mass, strength, and performance and to explore independent biomarkers for identifying older adults with low muscle strength.

**Methods:**

Ninety‐six older adults were selected from the Korean Frailty and Aging Cohort Study using stratified random sampling based on age and sex, and classified into four groups according to Asian Working Group for Sarcopenia 2019 criteria: normal (*n* = 25), low muscle mass (Low MM)‐only (*n* = 23), low muscle strength (Low MS)‐only (*n* = 25) and low physical performance (Low PP)‐only (*n* = 23). MiRNA profiles were generated through miRNA sequencing, and differentially expressed (DE) miRNAs among groups were identified using log2|Fold Change (FC)| ≥ 1 and a Benjamini–Hochberg (BH)‐adjusted *p* < 0.05. Subsequently, candidate miRNAs were validated by quantitative real‐time polymerase chain reaction. Differences in relative miRNA expression between groups were assessed using analysis of variance. The utility of identified miRNAs for discriminating older adults with low muscle strength was assessed using receiver operating characteristic (ROC) analysis.

**Results:**

In 96 older adults (50.0% women, mean age 76.6 ± 3.6 years), 16, 5 and 1 DE miRNAs were observed in comparisons of Low MS‐only vs. Low MM‐only, Low PP‐only vs. Low MM‐only, and Low PP‐only vs. Low MS‐only, respectively (log2|FC| ≥ 1 and BH‐adjusted *p* < 0.05). Among these, miR‐144‐3p, miR‐142‐3p and miR‐122‐3p overlapped across at least two comparisons. In the validation phase, miR‐144‐3p exhibited significantly higher expression in the Low MS‐only group than in other groups. Areas under the ROC curve (AUC) for miR‐144‐3p were 0.943 (95% CI = 0.854–1.000), 0.836 (95% CI = 0.698–0.974) and 0.844 (95% CI = 0.700–0.989) for distinguishing the Low MS‐only group from normal, Low MM‐only and Low PP‐only groups, respectively (*p* < 0.001). Kyoto Encyclopedia of Genes and Genomes analysis revealed that identified novel miRNAs were mainly associated with FoxO and insulin signalling (BH‐adjusted *p* < 0.001), with a trend toward neurotrophic signalling (BH‐adjusted *p* = 0.0647).

**Conclusions:**

miR‐144‐3p was identified as a novel biomarker for low muscle strength among older adults, independent of muscle mass and physical performance. Longitudinal studies are required to determine whether the identified miRNAs can function as predictive biomarkers for muscle strength decline.

## 1 Introduction

Sarcopenia refers to the progressive and generalized decline in skeletal muscle mass, muscle strength and physical performance with aging [1]. Since 1 October 2016, it has been classified as a disease under the International Classification of Diseases, 10th Revision, Clinical Modification (ICD‐10‐CM), with the code M62.84 [2]. Among the components of sarcopenia, maintaining muscle strength is a key factor in supporting healthy aging, as muscle strength has been recognized as the most reliable predictor of adverse health outcomes, including functional limitations, low quality of life and mortality [3, 4]. In particular, muscle strength has been proposed as a determinant of all‐cause mortality, ranging from apparently healthy populations to individuals with various acute and chronic conditions [5, 6]. As the clinical importance of muscle strength has been recognized, the revised European Working Group on Sarcopenia in Older People (EWGSOP2) guidelines highlighted muscle strength as a primary measure and recommended evaluating low muscle strength, along with low muscle quantity or quality, for the diagnosis of sarcopenia [7]. Recently, the newly introduced Global Leadership Initiative in Sarcopenia (GLIS) framework also emphasized the importance of muscle strength and muscle‐specific strength, defined as the ratio of muscle strength to muscle size, as well as muscle mass in the conceptual definition of sarcopenia [8]. The loss of muscle strength progresses much more rapidly than the concomitant loss of muscle mass in older adults. Even those who maintain or increase their muscle mass could not delay or prevent a decline in muscle strength [9]. This ultimately suggests the need to investigate why muscle strength declines irrespective of muscle mass. Consequently, it is crucial to understand the underlying cause of this decline by identifying potential biomarkers.

MicroRNAs (miRNAs) are a class of endogenous small noncoding RNA molecules with a mean length of 22 nucleotides. Most miRNAs are transcribed from DNA sequences into primary miRNAs, which are then processed into precursor miRNAs and ultimately into mature miRNAs [10]. These mature miRNAs primarily regulate the expression of target mRNAs by binding to the 3′ untranslated region [11]. They have been reported to regulate multiple biological processes, including cell growth and differentiation, development, metabolism and apoptosis [12]. Aberrant patterns in miRNA expression have been identified in various human diseases, including cardiovascular diseases, cancer, inflammatory diseases, neurodevelopmental disorders and other metabolic conditions; they are being further investigated as therapeutics or therapeutic targets for disease treatment [13]. In particular, abnormalities in miRNAs are involved in skeletal muscle development, leading to skeletal muscular diseases, such as Duchenne muscular dystrophy, dermatomyositis and other muscle‐related diseases [14].

Recently, miRNAs have been reported to play a significant role in age‐related alterations in skeletal muscle mass and function by regulating skeletal muscle cell proliferation and differentiation, apoptosis and senescence at the cellular level [15]. Importantly, miRNAs have been presented to be differentially expressed in both muscle atrophy‐induced mice and old mice, as well as being associated with skeletal muscle wasting in older adults, suggesting their systemic role in age‐related muscle atrophy [16, 17, 18]. In particular, several circulating miRNAs have been identified as being associated with sarcopenia in community‐dwelling older adults, implicating their role as potential therapeutic targets for sarcopenia [19]. Millet et al. recently presented the circulating miRNA signatures associated with sarcopenia in older adults [20]. In addition, myo‐related miRNAs (miR‐486) and inflammation‐related miRNAs (miR‐146a) have been reported to be more effective than inflammatory markers in detecting sarcopenia [21]. These previous studies emphasized miRNAs as potential biomarkers for sarcopenia detection.

To date, previous studies have comprehensively examined the role of miRNAs in detecting sarcopenia itself; however, the miRNA profiles associated with individual components of sarcopenia remain insufficiently characterized. Few clinical studies have focused on muscle strength‐specific miRNAs in community‐dwelling older adults. Zhang et al. previously reported that resistance training altered muscle‐specific miRNAs in the muscles and plasma of older adults and that these alterations may partially explain the changes in muscle strength induced by training. Specifically, changes in knee extensor strength with resistance training were strongly correlated with changes in miR‐133a, miR‐133b, miR‐206 and miR‐499 in the plasma or muscle tissue of older adults [22]. Furthermore, Mitchell et al. identified that muscle‐specific miRNAs, such as miR‐100‐5p, miR‐99b‐5p and miR‐191‐5p, positively correlated with isokinetic knee extension strength in middle‐aged men [23]. However, these previous studies did not consider other muscle‐related parameters, such as muscle mass or physical performance; thus, whether the identified miRNAs were exclusively associated with muscle strength could not be determined. This underscores an important gap in the current literature regarding the independent association between circulating miRNAs and muscle strength. Therefore, to independently detect declines in muscle strength, our study aimed to compare the miRNA profiles in four distinct groups of older adults comprising normal, low muscle mass only, low muscle strength only and low physical performance only groups. We achieved this by comprehensively screening and validating muscle strength‐specific miRNA candidates.

## 2 Materials and Methods

### 2.1 Study Participants

The Korean Frailty and Aging Cohort Study (KFACS) is an ongoing multicentre cohort study that enrolled participants from 10 centres, including eight hospitals and two public health centres, across urban and rural regions in the Republic of Korea. The baseline survey was conducted between May 2016 and November 2017, with subsequent follow‐up assessments scheduled at two‐year intervals. A total of 3014 participants were recruited during the baseline phase from sex‐ and age‐stratified samples of community‐dwelling older adults aged 70–84 years, across 10 study centres located in urban, suburban and rural regions in the Republic of Korea. To reduce the risk of selection bias, the participants were enrolled from diverse settings, such as senior welfare centres, public health centres, residential complexes, apartments and outpatient clinics.^S1^ In the baseline survey, all clinical assessments, including in‐person interviews, health examinations and laboratory measures, were conducted by clinical research coordinators at the clinical site of each study centre. To ensure standardized assessments across all sites, clinical research coordinators at each site were trained according to the KFACS protocol and data entry guidelines.^S1^

**FIGURE 1:**
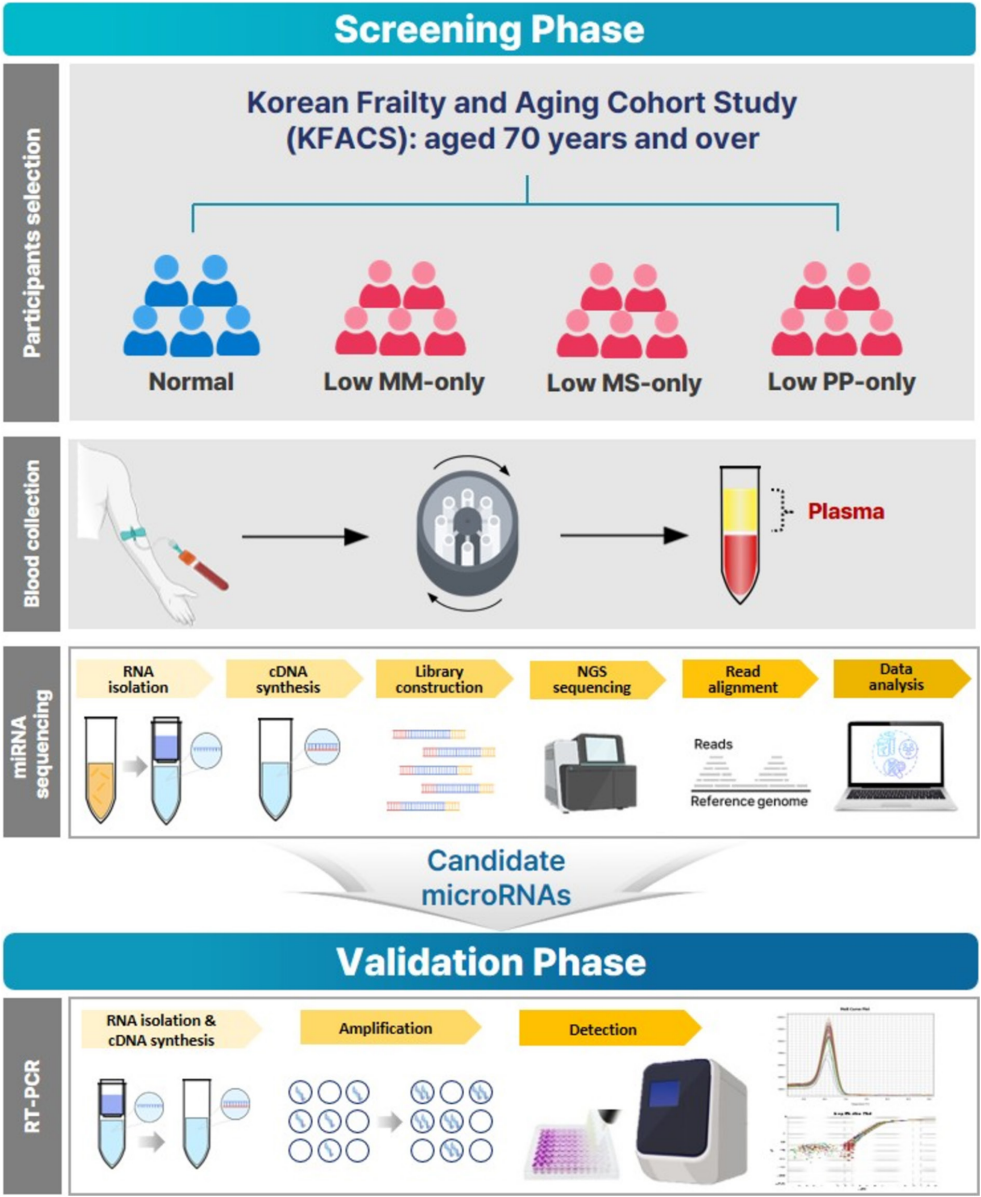
A schematic diagram with an overview of the study design.

Body composition was measured using two methods: bioelectrical impedance analysis (BIA) at two public health centres (*n* = 611) and dual‐energy X‐ray absorptiometry (DXA) at eight hospital centres (*n* = 2403). Owing to a systematic bias identified in the measurement of appendicular lean mass between the BIA and DXA measures, only participants whose body composition was assessed using DXA were included in our analysis.^S2‐S4^ An overview of the study design is presented as a schematic diagram in Figure 1. In addition, we applied the following exclusion criteria to minimize potential confounding factors that could affect small ncRNA expression and sarcopenia based on prior studies [21, 24, 25]. Participants with uncontrolled diabetes, dyslipidaemia, myocardial infarction, congestive heart failure, angina pectoris, peripheral vascular disease, cerebrovascular disease, chronic obstructive pulmonary disease, cancer, liver disease, kidney disease, osteoarthritis and rheumatoid arthritis were excluded. These comorbidities were defined based on a self‐reported history of physician‐diagnosed diseases, obtained through standardized questionnaires at baseline. Those with neurological dysfunction, including hemiplegia, paraplegia or Parkinson's disease; those who drank alcohol more than 4 days/week or smoked every day; and those who had artificial joints, pins, plates, metal suture materials or other types of metal objects in the appendicular body regions were also excluded. Consequently, a total of 994 older adults were eligible for our study.

Study participants were categorized into the following groups based on the AWGS 2019 criteria for low muscle mass, low muscle strength and low physical performance: ‘normal’, ‘low muscle mass (Low MM)‐only’, ‘low muscle strength (Low MS)‐only’ and ‘low physical performance (Low PP)‐only’. The detailed operational definitions of the groups were as follows:

- **Normal:** Appendicular skeletal muscle mass (ASM) index of ≥ 7.0 kg/m^2^ for men and ≥ 5.4 kg/m^2^ for women; handgrip strength of ≥ 28 kg for men and ≥ 18 kg for women; and gait speed of ≥ 1.0 m/s for men and women.
- **Low MM‐only:** ASM index of < 7.0 kg/m^2^ for men and < 5.4 kg/m^2^ for women; handgrip strength of ≥28 kg for men and ≥ 18 kg for women; and gait speed of ≥ 1.0 m/s for men and women.
- **Low MS‐only:** ASM index of ≥ 7.0 kg/m^2^ for men and ≥ 5.4 kg/m^2^ for women; handgrip strength of < 28 kg for men and < 18 kg for women; and gait speed of ≥ 1.0 m/s for men and women.
- **Low PP‐only:** ASM index of ≥ 7.0 kg/m^2^ for men and ≥ 5.4 kg/m^2^ for women; handgrip strength of ≥ 28 kg for men and ≥ 18 kg for women; and gait speed of < 1.0 m/s for men and women.

**FIGURE 2:**
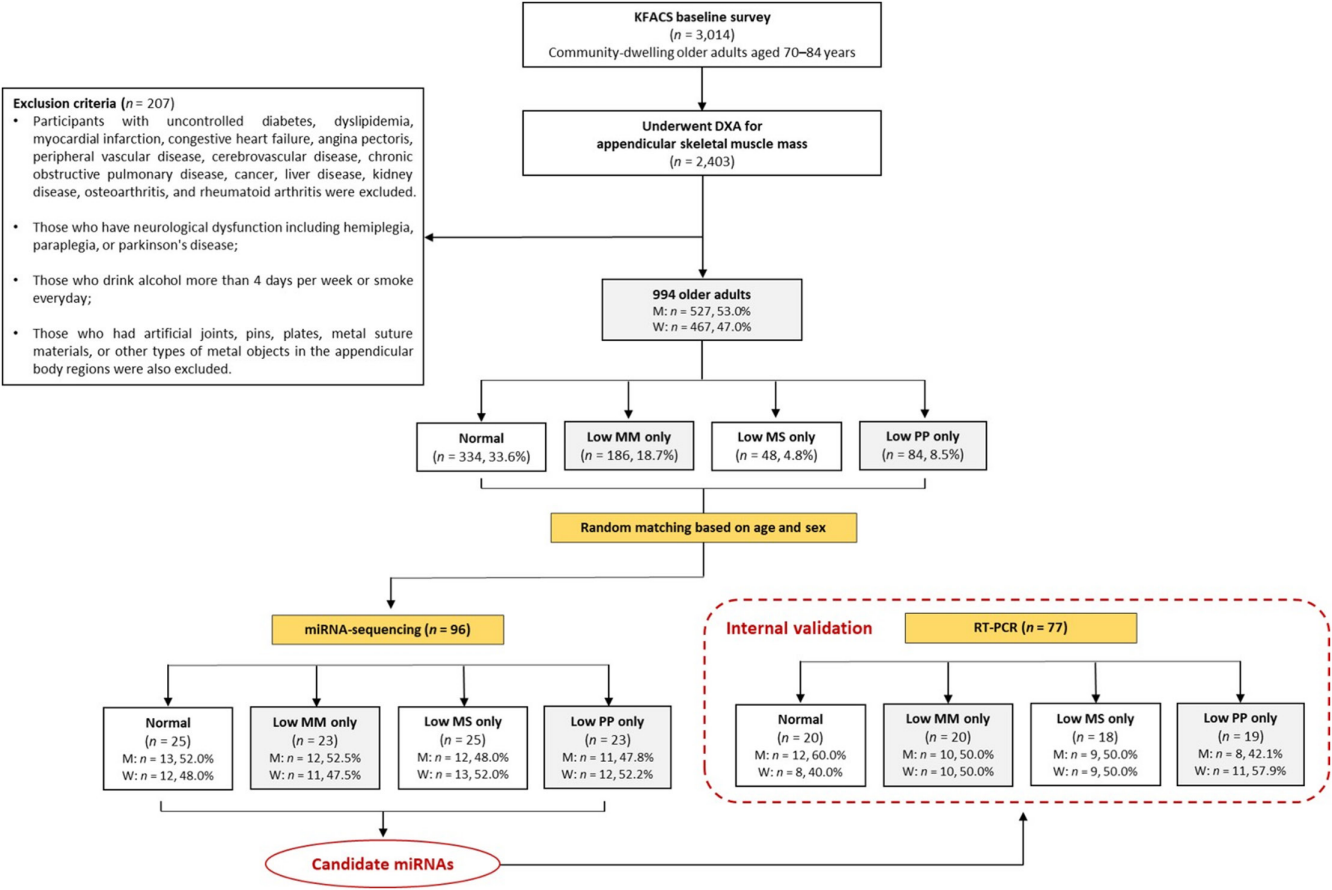
Flowchart of study participants.

The study included 334 older adults in the normal group, 186 in the low MM‐only group, 48 in the low MS‐only group and 84 in the low PP‐only group. Among them, 96 older adults were selected, with 23 to 25 participants assigned to each group to detect differentially expressed miRNAs across groups, and the sample size was estimated based on previous miRNA profiling studies.^20,S5^ To ensure the homogeneity of the study groups, stratified random sampling based on age and sex was applied when selecting participants for miRNA sequencing. Random numbers were generated with the RAND() function in Excel to maintain unbiased and balanced group allocation. Subsequently, a total of 77 older adults were selected from the 96 older adults for quantitative real‐time polymerase chain reaction (RT‐PCR) analysis, with approximately 20 participants per group. Some participants who had insufficient plasma samples for RT‐PCR were excluded (*n* = 19). Nevertheless, the characteristics of those included in both the miRNA sequencing and RT‐PCR analyses remained consistent. A detailed flowchart outlining the selection and exclusion processes for the study participants is presented in Figure 2. Blinding was ensured between the personnel responsible for group allocation and those responsible for data acquisition and analysis, with all analyses conducted using anonymized identification numbers. This study was conducted in accordance with the STROBE (Strengthening the Reporting of Observational Studies in Epidemiology) guidelines and was conducted in accordance with the ethical standards laid down in the 1964 Declaration of Helsinki. The KFACS protocol was reviewed and approved by the Clinical Research Ethics Committee of Kyung Hee University Medical Center (IRB No. 2015‐12‐103). Approval for the present study was also obtained from this committee (IRB No. 2022‐08‐047).

### 2.2 Sarcopenia Components

ASM was measured using DXA (Hologic DXA; Hologic Inc., Bedford, MA, USA, and Lunar; GE Healthcare, Madison, WI, USA) by summing the lean masses of both arms and legs (kg). The ASM index was calculated as the ASM divided by the height squared (ASM/height^2^). Assessment of 40 volunteers in our laboratory, with repositioning between scans, revealed that the coefficients of variation for appendicular lean mass (sum of the lean mass of the arms and legs) were consistently below 2.5%.^S6^ Handgrip strength was evaluated twice for each hand using a digital dynamometer (T.K.K.5401; Takei Scientific Instruments Co. Ltd., Tokyo, Japan) in accordance with standardized measurement protocols, and the highest value was used for analysis.^S7^ The reliability of the Takei handgrip dynamometer has been reported in a previous study, with intraclass correlation coefficients ranging from 0.898 to 0.948, indicating an almost perfect correlation.^S8^ The usual gait speed was measured twice over a 4‐m course using an automatic timer, with 1.5 m allocated for acceleration and deceleration at the start and end of the course, and the average of the two trials was recorded (Gaitspeedmeter, Dynamicphysiology, Daejeon, Korea).^S9^ To define low physical performance, we used gait speed alone, as other physical performance measures such as the five‐times sit‐to‐stand test and the Short Physical Performance Battery (SPPB) commonly include the chair stand movement. According to the EWGSOP2 criteria, the chair stand test has been reported as a proxy measure of lower extremity muscle strength.^S10^ To ensure a more specific and conventional assessment of mobility‐related physical performance, we adopted gait speed as the primary measure, as it better reflects functional capacity independent of muscle strength.

### 2.3 Blood Sample Collection

The participants were instructed to avoid taking any medications before blood collection, which was conducted from the antecubital vein at 7:30–8:00 AM, following an overnight fast. Blood samples were placed in ethylenediaminetetraacetic acid (EDTA) tubes and maintained upright at 2°C–8°C. Plasma was separated by centrifuging the samples at 3000 rpm for 10 min and then promptly storing them at about −70°C until processing. Plasma was used for miRNA analysis as it better preserves the endogenous circulating microRNA profiles and minimizes pre‐analytical variability that may arise during the coagulation process required for serum collection.^S11^ The biospecimens and data used in this study were provided by the Biobank of Kyung Hee University Hospital (2024‐1).

### 2.4 MicroRNAs Sequencing and Data Analysis

According to the manufacturer's protocol, total RNA was extracted using the Qiagen miRNeasy Serum/Plasma Kit (Catalogue no. 217184) and converted into cDNA. To subsequently construct sequencing libraries, the SMARTer smRNA‐Seq Kit for Illumina (TAKARA) was used. The constructed libraries were validated by assessing their size, purity, and concentration using an Agilent Bioanalyzer. The libraries were pooled in equimolar amounts and sequenced using an Illumina NovaSeq instrument. Library quality was evaluated using the Illumina pipeline modules.

Raw reads of small RNAs were preprocessed to remove adapter sequences using the Cutadapt program. Short sequences were aligned to the human reference genome to quantify known and novel miRNAs using the miRDeep2 software program. Log Counts Per Million and log fold change (FC) were calculated among groups. To test the statistical hypothesis, the exact test was performed among groups using edgeR. Differentially expressed miRNAs (DE miRNAs) among groups were determined using ExDEGA version 5.1.1.4 software (eBiogen, Seoul, Korea), with a log_2_|FC| ≥ 1 and an adjusted *p*‐value < 0.05 using Benjamini–Hochberg (BH) correction. The log_2_|FC| threshold of 1 was selected to balance the identification of biologically meaningful expression changes while retaining a sufficient number of candidate miRNAs for validation analysis.^S12^ Although higher thresholds (e.g., log_2_|FC| ≥ 2) may capture more pronounced differences, they can substantially limit the number of detectable candidates. This criterion is also commonly adopted in previous studies evaluating miRNAs as disease biomarkers.^S13‐S15^

### 2.5 RT‐PCR for Validation

Reverse transcription was performed using the miRCURY LNA RT Kit (Qiagen, Germany), according to the manufacturer's instructions. cDNA of microRNA was amplified using the miRCURY LNA miRNA PCR Primers as follows: miR‐101‐3p (YP00204786), miR‐122‐3p (YP00204437), miR‐142‐3p (YP00204291), miR‐144‐3p (YP00204754), miR‐1277‐3p (YP02114217), miR23a‐5p (YP00205631), miR‐29a‐5p (YP00204430), miR‐1‐5p (YP02103541), miR‐210‐3p (YP00204333), miR‐133a‐3p (YP00204788), miR‐424‐5p (YP00204736) and for reference controls cel_miR‐39‐3p (YP00203952) (Qiagen, Germany). Real‐time PCR was performed on a QuantStudio 1 Real‐Time PCR System (Applied Biosystems, USA) using the miRCURY SYBR Green PCR Kit (Qiagen, Germany) according to the manufacturer's instructions. Thermal cycling conditions were as follows: 95°C for 2 min, followed by 40 cycles at 95°C for 10 s and 56°C for 1 min. Quantification was performed using QuantStudio software v1.5.2 (Applied Biosystems). Relative miRNA expression levels were calculated using the 2^−ΔΔCT^ method, with normalization to cel‐miR‐39‐3p. Differences in relative miRNA expression levels between groups were assessed using one‐way analysis of variance (ANOVA).

To analyse the utility of miRNAs identified through the screening and validation phase in discriminating the declines in muscle strength, receiver operating characteristic (ROC) curve analysis was performed to calculate the area under the curve (AUC).

## 3 Results

### 3.1 Clinical Characteristics of Study Participants

Demographic characteristics of the study participants, classified into ‘Normal’, ‘Low MM‐only’, ‘Low MS‐only’ and ‘Low PP‐only’ groups were shown in Table 1. The participants had an average age of 76.6 ± 3.6 years, and 50% were women. As anticipated, appendicular lean mass index, handgrip strength and physical performance measures, such as usual gait speed, five‐times sit‐to‐stand times, timed ‘Up & Go’, and Short Physical Performance Battery score, significantly differed among the four groups (all, *p* < 0.05). No significant differences in physical activity, nutritional status or haematological variables were observed among the four groups (all, *p* ≥ 0.05).

**TABLE 1:** Demographic characteristics of study participants categorized by muscle mass, muscle strength and physical performance status.

Variable	Group (*n* = 96)										
	Overall		Normal		Low MM‐only		Low MS‐only		Low PP‐only		*p*
	(*n* = 96)		(*n* = 25)		(*n* = 23)		(*n* = 25)		(*n* = 23)		
Age (years)	76.6	±3.6	77.4	±3.5	77.1	±3.7	76.7	±4.3	77.5	±2.9	0.878
Sex (women)	48	(50.0)	12	(48.0)	11	(47.5)	13	(52.0)	12	(52.2)	0.983
Low physical activity	8	(8.3)	0	(0.0)	3	(13.0)	4	(16.7)	1	(4.3)	0.127
Malnutrition	5	(5.2)	1	(4.0)	1	(4.0)	0	(0.0)	3	(13.0)	0.230
BMI (kg/m^2^)	24.0	±2.6	24.4	±2.2^a^	22.3	±1.9^a,d,e^	24.2	±2.2^d^	25.2	±3.1^e^	< 0.001
BMI (≥ 23 kg/m^2^)	62	(64.6)	20	(80.0)	8	(34.8)	17	(68.0)	17	(73.9)	0.005
Waist circumference (≥ 90 cm in men and ≥ 85 cm in women)	51	(53.1)	18	(72.0)	7	(30.4)	13	(52.0)	13	(56.5)	0.037
Appendicular lean mass (kg)											
Men	20.0	±2.1	21.0	±2.3^a^	17.9	±1.4^a,d,e^	20.3	±1.7^d^	20.7	±1.6^e^	< 0.001
Women	13.3	±2.0	14.3	±2.1^a^	11.1	±0.8^a,d,e^	13.5	±1.0^d^	14.3	±1.8^e^	< 0.001
Appendicular lean mass/height^2^ (kg/m^2^)											
Men	7.3	±0.6	7.5	±0.4^a^	6.5	±0.4^a,d,e^	7.6	±0.5^d^	7.7	±0.3^e^	< 0.001
Women	5.9	± 0.8	6.3	± 0.6^a^	5.0	± 0.4^a,d,e^	5.8	±0.4^d^	6.3	±0.8^e^	< 0.001
Percentage body fat (%)											
Men	25.4	±6.1	25.0	±6.1	28.2	±5.0	23.1	±5.9	25.7	±6.9	0.257
Women	35.6	±5.2	35.9	±2.8	36.4	±5.7	35.6	±5.8	34.6	±6.3	0.869
Physical performance											
Short Physical Performance Battery score	11.0	±1.2	11.1	±1.1	11.6	±0.7^e^	11.0	±1.1	10.4	±1.4^e^	0.004
Timed ‘Up & Go’ (s)	10.1	±2.0	9.4	±1.9^c^	9.3	±1.3^e^	10.4	±1.7	11.4	±2.3^c,e^	< 0.001
Hand grip strength (kg)	26.5	±8.1	29.5	±8.1^b^	27.7	±7.5^d^	20.4	±4.8^b,d,f^	28.7	±8.3^f^	< 0.001
Five‐times sit‐to‐stand (s)*	11.0	±3.2	10.4	±0.3.1	9.7	±2.0	11.2	±2.9	12.88	±4.1	0.005
Usual Gait speed (m/s)	1.2	±0.2	1.2	±0.3^c^	1.2	±0.1^e^	1.2	±0.2^f^	0.9	±0.1^c,e,f^	< 0.001
Haematological											
Albumin (g/dL)	4.3	±0.2	4.3	±0.2	4.4	±0.3	4.3	±0.2	4.3	±0.2	0.128
Total cholesterol (mg/dL)	184.3	±35.0	189.3	±28.1	182.4	±40.2	187.0	±31.9	177.7	±40.3	0.679
HDL‐cholesterol (mg/dL)	50.8	±13.2	49.9	±11.7	53.9	±16.5	48.9	±12.7	50.8	±11.9	0.600
LDL‐cholesterol (mg/dL)	120.1	±32.2	125.2	±28.0	116.9	±33.4	127.1	±31.1	110.4	±35.3	0.251
Triglyceride (mg/dL)	116.0	±51.9	126.6	±65.6	110.6	±48.9	106.6	±33.2	120.1	±55.5	0.527
High‐sensitivity C‐reactive protein*	1.3	±2.2	1.3	±1.7	1.2	±1.7	1.5	±3.5	1.2	±1.3	0.968
Haemoglobin (g/dL)	13.4	±1.2	13.4	±1.4	13.7	±1.5	13.4	±1.1	13.2	±1.0	0.655
Glycosylated haemoglobin (%)	5.9	±0.7	5.8	±0.4	5.9	±0.8	5.9	±0.5	5.9	±0.9	0.897
HOMA‐IR*	1.6	±1.2	1.8	±1.0	1.4	±0.7	1.4	±0.8	2.0	±1.8	0.147
Creatinine (mg/dL)	0.8	±0.2	0.8	±0.2	0.9	±0.2	0.8	±0.2	0.9	±0.3	0.186
25‐hydroxy vitamin D (ng/mL)	23.1	±9.1	22.3	±7.5	25.0	±8.3	19.7	±8.4	25.9	±11.2	0.079

*Note:* Values are means (± SD) or numbers (%). The *p*‐values were calculated by analysis of variance (ANOVA) for continuous variables and chi‐square or Fisher's exact test for categorical variables. Superscript letters indicate statistically significant differences (*p* < 0.05) in post hoc comparisons between groups as follows: ^a^Normal vs. Low MM‐only, ^b^Normal vs. Low MS‐only, ^c^Normal vs. Low PP‐only, ^d^Low MM‐only vs. Low MS‐only, ^e^Low MM‐only vs. Low PP‐only, ^f^Low MS‐only vs. Low PP‐only.

Abbreviations: BMI, body mass index; HOMA‐IR, homeostasis model assessment for insulin resistance.

* Some data were missing.

### 3.2 Overlapped Differentially Expressed miRNAs in the Plasma of Low MM‐only, Low MS‐only, and Low PP‐Only Groups

**FIGURE 3:**
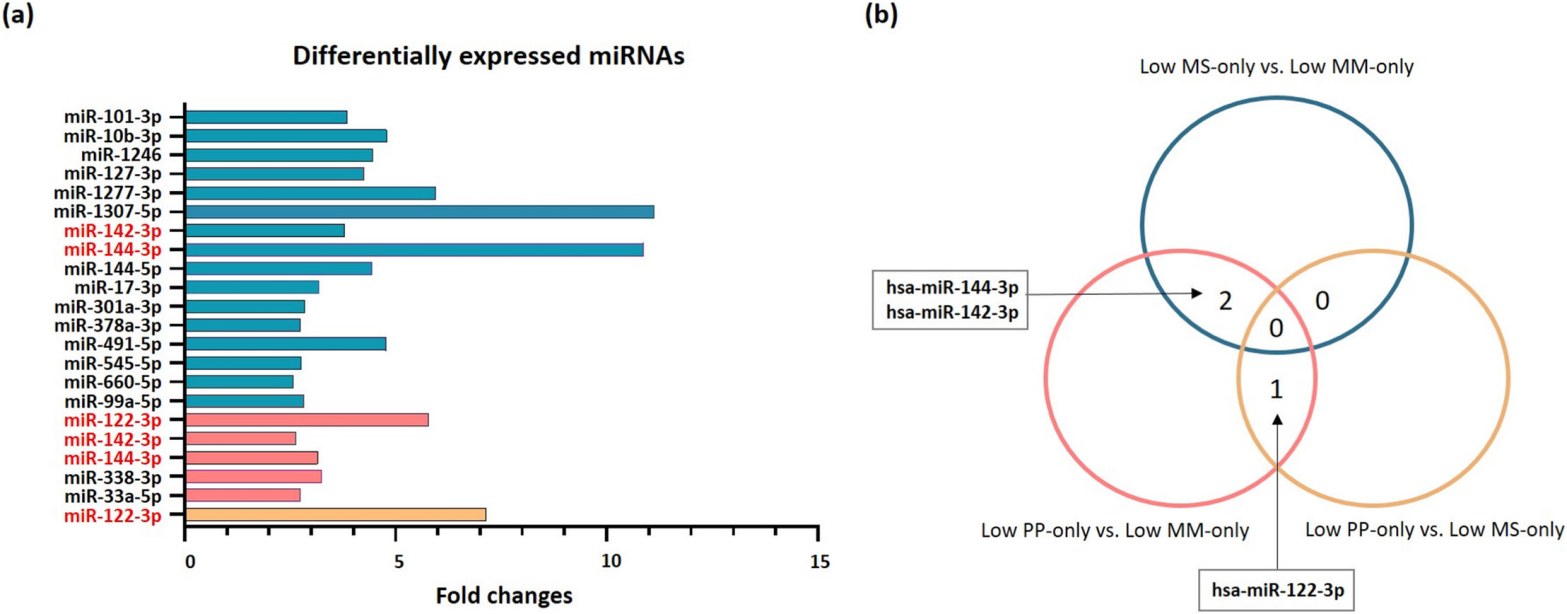
(a) Differentially expressed miRNAs in plasma of LowMM‐only, LowMS‐only and LowPP‐only groups (log2|FC| ≥ 1 and BH adjusted *p*‐value < 0.05); (b) Venn diagram of overlapped differentially expressed miRNAs in plasma of LowMM‐only, LowMS‐only and LowPP‐only. *Abbreviations:* MM, muscle mass; MS, muscle strength; PP, physical performance. Blue bar indicates LowMS‐only vs. LowMM‐only, red bar indicates LowPP‐only vs. LowMM‐only, and yellow bar indicated LowPP‐only vs. LowMS‐only.

miRNA expression levels of each sarcopenia component were examined to determine their independence. DE miRNAs with log2|FC| ≥ 1 and BH‐adjusted *p* < 0.05 were analysed across group comparisons: Low MS‐only versus (vs.) Low MM‐only, Low PP‐only vs. Low MM‐only and Low PP‐only vs. Low MS‐only. A comprehensive list of miRNAs sequenced in the library, reads per million (RPM), and FC in the plasma of each comparison group was presented in Tables S1–S3. We identified 16, 5 and 1 DE miRNAs in comparisons of Low MS‐only vs. Low MM‐only, Low PP‐only vs. Low MM‐only and Low PP‐only vs. Low MS‐only, respectively (Figure 3a). Among the DE miRNAs identified, three (miR‐144‐3p, miR‐142‐3p and miR‐122‐3p) overlapped in at least two group comparisons. Specifically, differential expression of miR‐144‐3p and miR‐142‐3p was observed in both Low MS‐only vs. Low MM‐only and Low PP‐only vs. Low MM‐only comparisons. Additionally, miR‐122‐3p was differentially expressed in comparisons of both the Low PP‐only vs. Low MM‐only and Low PP‐only vs. Low MS‐only (Figure 3b).

### 3.3 Validation of Candidate miRNAs Using RT‐PCR

**FIGURE 4:**
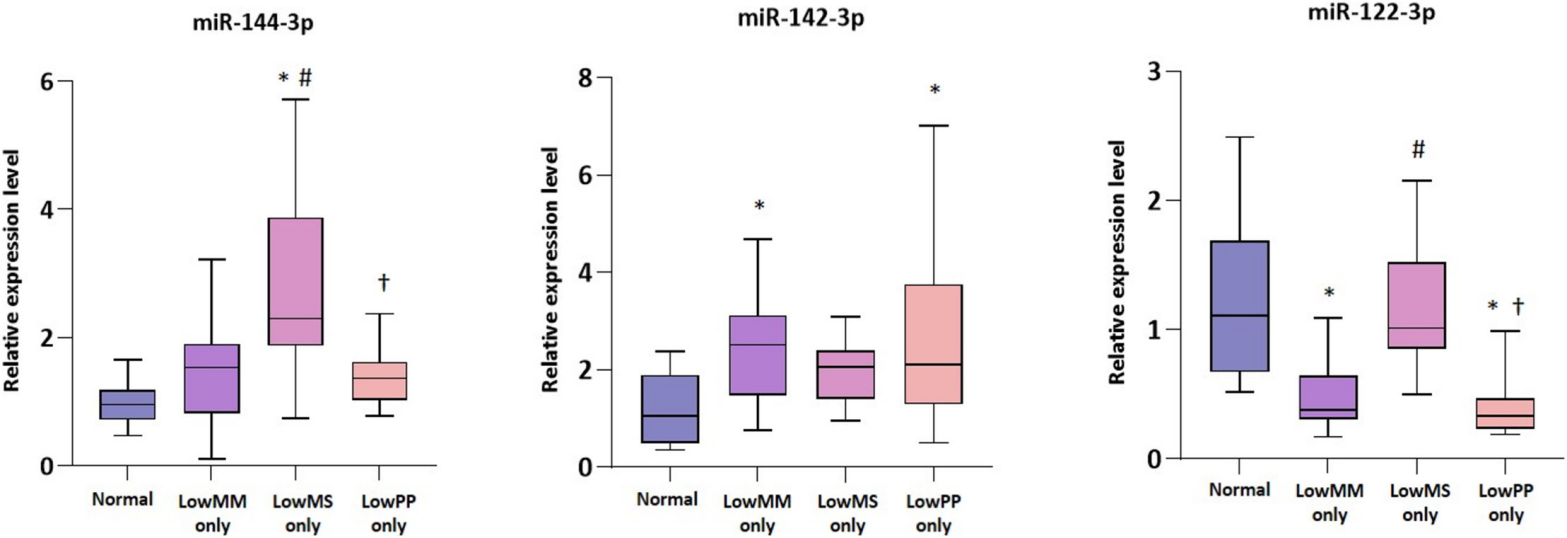
Validation of differentially expressed miRNAs using RT‐PCR. *Abbreviations:* MM, muscle mass; MS, muscle strength; PP, physical performance; NS, not significant. *p < 0.05 vs. Control; ^#^ *p* < 0.05 vs. LowMM‐only; ^†^ *p* < 0.05 vs. LowMS‐only.

From the miRNA sequencing, three miRNAs (miR‐144‐3p, miR‐142‐3p and miR‐122‐3p) were selected as candidates for validation through quantitative RT‐PCR. The expression levels of miR‐144‐3p and miR‐122‐3p were significantly elevated in the Low MS‐only group compared to the other groups (all, *p* < 0.05), although miR‐122‐3p levels did not differ significantly from those in the normal group. Furthermore, miR‐142‐3p expression was significantly higher in the Low MM‐only and Low PP‐only groups than in the normal group (Figure 4).

**FIGURE 5:**
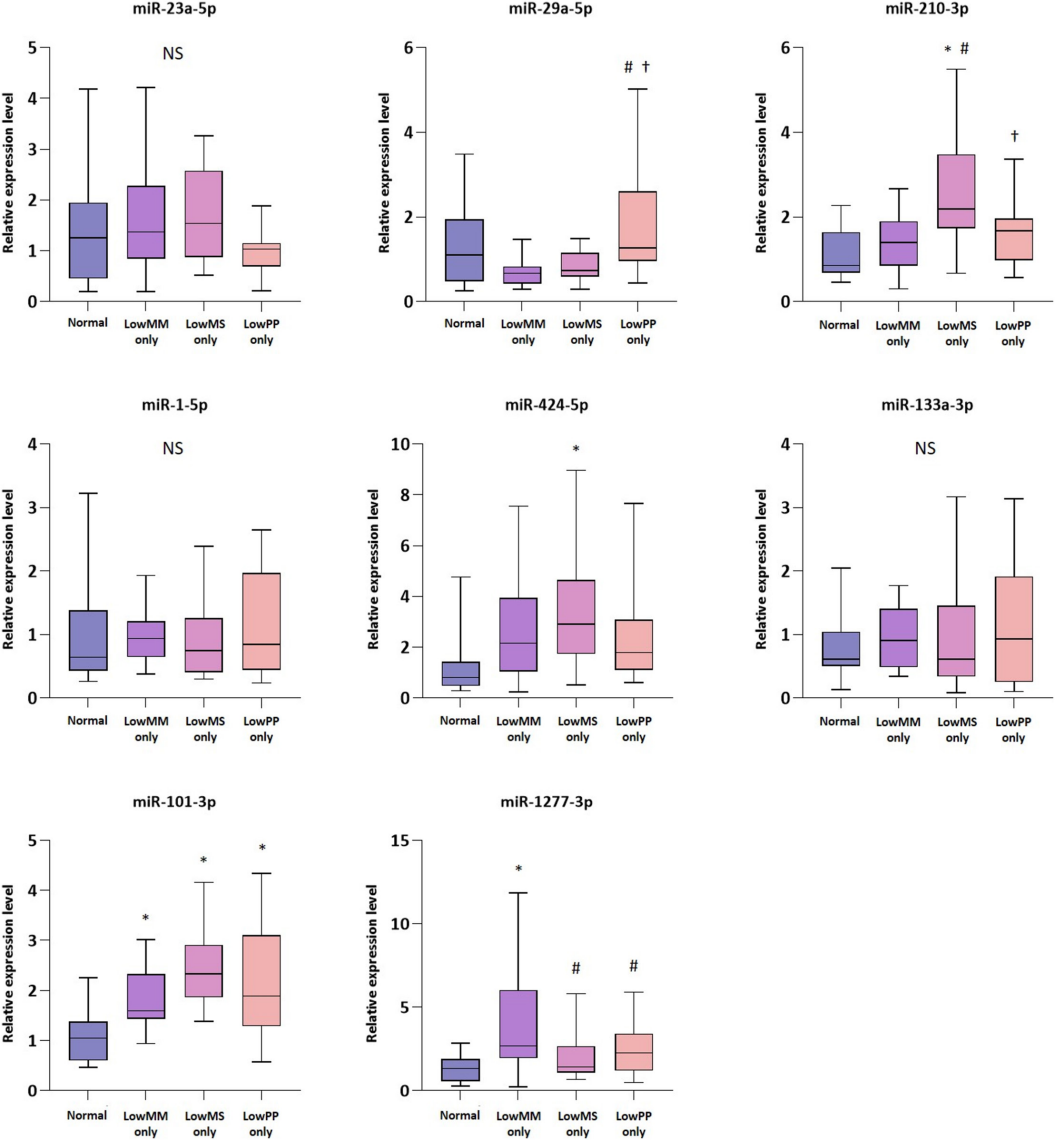
Validation of candidate miRNAs associated with sarcopenia using RT‐PCR. *Abbreviations:* MM, muscle mass; MS, muscle strength; PP, physical performance; NS, not significant. *p < 0.05 vs. Control; ^#^ *p* < 0.05 vs. LowMM‐only; ^†^ *p* < 0.05 vs. LowMS‐only.

In addition, eight miRNAs (miR‐23a, miR‐29a‐5p, miR‐210‐3p, miR‐1‐5p, miR‐424‐5p, miR‐133a‐3p, miR‐101‐3p and miR‐1277‐3p) identified in our previous systematic review as being associated with sarcopenia, frailty or both [26] were also included in the RT‐PCR analysis. The expression levels of miR‐210‐3p were significantly elevated in the Low MS‐only group compared to those in the other groups (all, *p* < 0.05). miR‐29a‐5p expression was significantly higher in the Low PP‐only group than in the Low MM‐only and Low MS‐only groups (*p* < 0.05), but did not differ from the normal group. Furthermore, miR‐424‐5p was significantly elevated in the Low MS‐only group compared to the normal group, and miR‐101‐3p was significantly elevated in the Low MM‐only, Low MS‐only and Low PP‐only groups, all compared to the normal group (*p* < 0.05). Meanwhile, miR‐1277‐3p was significantly elevated in the Low MM‐only group compared to the normal group (*p* < 0.05), whereas it was lower in the Low MS‐only and Low PP‐only groups compared to the Low MM‐only group. No significant differences were detected in the expression levels of miR‐23a‐5p, miR‐1‐5p or miR‐133a‐3p across the groups (Figure 5).

A subgroup analysis stratified by sex revealed that miR‐144‐3p expression was significantly higher in the Low MS‐only group compared to the other groups in both older men and women (*p* < 0.05). In contrast, miR‐210‐3p expression was significantly elevated in the Low MS‐only group compared to the other groups in older men but not older women.

**FIGURE 6:**
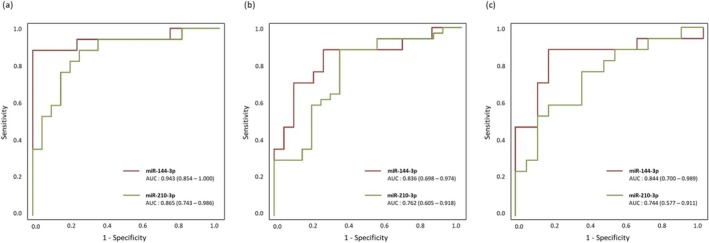
Receiver operating characteristic (ROC) analysis of miR‐144‐3p and miR‐210‐3p for detecting the Low MS‐only group, compared with (a) the Normal group, (b) the Low MM‐only group, and (c) the Low PP‐only group.

ROC curve analysis was conducted to assess whether miR‐144‐3p and miR‐210‐3p, which were significantly elevated in the Low MS‐only group, could effectively discriminate the Low MS‐only group from the normal, Low MM‐only and Low PP‐only groups. The AUC for miR‐144‐3p and miR‐210‐3p was 0.943 (95% CI = 0.854–1.000) and 0.865 (95% CI = 0.743–0.986), respectively, in distinguishing the Low MS‐only group from the normal group (Figure 6a). In addition, the AUC for miR‐144‐3p and miR‐210‐3p was 0.836 (95% CI = 0.698–0.974) and 0.762 (95% CI = 0.605–0.918), respectively, in distinguishing the Low MS‐only group from the Low MM‐only group (Figure 6b). To differentiate the Low MS‐only group from the Low PP‐only group, the AUC for miR‐144‐3p and miR‐210‐3p was 0.844 (95% CI = 0.700–0.989) and 0.744 (95% CI = 0.577–0.911), respectively (Figure 6c).

### 3.4 Possible Underlying Mechanism via Functional Enrichment

We identified that two miRNAs, miR‐144‐3p and miR‐210‐3p, were independently associated with low muscle strength. To examine target genes and their biological functions, miRWalk Version 3.0 was used. Specifically, 235 genes were predicted to be targets of miR‐144‐3p, and 4540 genes were predicted to be targets of miR‐210‐3p (data not shown). Kyoto Encyclopedia of Genes and Genomes (KEGG) pathway enrichment analysis identified the top 10 significantly enriched pathways associated with the two muscle strength‐specific miRNAs. Interestingly, miR‐144‐3p was primarily involved in FoxO and insulin signalling (BH‐adjusted *p* < 0.001) and exhibited a borderline association with the neurotrophic signalling pathway (BH‐adjusted *p* = 0.0647). However, no significant pathways were identified for miR‐210‐3p (Table 2).

**TABLE 2:** The top 10 most significantly enriched pathways of muscle strength‐specific miRNAs identified in the plasma of older adults.

Name	*p*	BH adjusted *p*
miR‐144‐3p		
*KEGG*		
FoxO signalling	< 0.001	< 0.001
Insulin signalling	< 0.001	< 0.001
Neurotrophin signalling	0.001	0.0647
Progesterone‐mediated oocyte maturation	0.005	0.2425
Relaxin signalling	0.010	0.3233
Pancreatic cancer	0.013	0.3233
Cushing syndrome	0.018	0.3233
Colorectal cancer	0.018	0.3233
mTOR signalling	0.019	0.3233
Hippo signalling	0.019	0.3233
miR‐210‐3p		
*KEGG*		
Autophagy	0.007	0.9308
Adherence junction	0.009	0.9308
FoxO signalling pathway	0.010	0.9308
Biosynthesis of unsaturated fatty acids	0.011	0.9308
Endocytosis	0.029	0.9308
Oxytocin signalling pathway	0.031	0.9308
Axon guidance	0.032	0.9308
Morphine addiction	0.040	0.9308
Regulation of actin_cytoskeleton	0.041	0.9308
Adrenergic signalling in cardiomyocytes	0.046	0.9308

Abbreviation: KEGG, Kyoto Encyclopedia of Genes and Genomes.

## 4 Discussion

Studies identifying biomarkers that independently detect sarcopenia components, such as muscle mass, muscle strength and physical performance, remain scarce. However, prior studies have reported associations of circulating miRNA expression levels with sarcopenia. In this context, this study is the first to elucidate miRNAs independently associated with muscle mass, muscle strength and physical performance, as well as to confirm their statistically independent significance across these components. Through the screening and validation phases, we identified miR‐144‐3p, as a novel miRNA, associated with low muscle strength. Additionally, miR‐210‐3p, a candidate miRNA associated with sarcopenia previously reported in our systematic review [26], was confirmed to be related to declines in muscle strength. Therefore, this study holds clinical significance by identifying novel miRNA for the detection of low muscle strength in older adults.

We identified differentially expressed miRNAs in each group (muscle mass, muscle strength and physical performance) and demonstrated through comparative analysis that miRNA signatures exhibited distinct patterns associated with the individual components of sarcopenia. These findings underscore the need to consider not only sarcopenia as a whole but also its individual components in the identification of sarcopenia biomarkers. Muscle strength has been reported to decline to a greater extent than muscle mass in older adults, indicating that muscle mass and strength are independently regulated during the aging process [9]. Jung et al. observed a weak correlation between miRNA expression profiles in aged mice and those in a disuse atrophy model, suggesting that age‐related muscle atrophy and disuse‐induced atrophy may originate from different mechanisms [18]. Mitchell et al. identified distinct patterns where muscle strength exhibited significant positive correlations with miR‐100‐5p, miR‐99b‐5p, and miR‐191‐5p, whereas cross‐sectional area (CSA) was correlated only with miR‐99b‐5p, reporting an inconsistency between two muscle‐related parameters [23]. Liu et al. also presented that skeletal muscle mass index positively correlated with miR‐486 and miR‐146a, while grip strength was exclusively associated with miR‐146a. In contrast, gait speed showed no significant correlations with miRNAs [21]. The divergence in the miRNA associations among sarcopenia components implies that these miRNAs may regulate muscle size and function via different pathways [23]. Furthermore, Wardle et al. identified distinct miRNA expression patterns between endurance and resistance exercises. Differences in miRNA expression by exercise type have been proposed to reflect their potential involvement in skeletal muscle adaptations related to muscle strength and physical function rather than changes in muscle mass [27]. In light of these previous studies, our results corroborate that muscle mass, muscle strength and physical performance are interrelated while also functioning as independent entities. Accordingly, identifying independent biomarkers for each component of sarcopenia, along with their associated mechanisms, will facilitate the early detection and prevention of its decline, ultimately addressing the multifaceted nature of sarcopenia.

Our finding that miR‐144‐3p and miR‐210‐3p are independently associated with low muscle strength does not align with previous studies reporting an association with muscle strength [21, 22, 23]. These discrepancies may be attributed to the consideration of other muscle‐related indicators, such as muscle mass and physical performance. While previous studies have identified the association of miRNAs with muscle strength or its changes without accounting for other muscle‐related indicators, our study identified miRNAs in older adults with low muscle strength but normal muscle mass and physical performance. This approach facilitated the identification of an independent association between muscle strength and miRNA expression. A previous study defined the dynapenia group as older adults with normal muscle mass but reduced muscle strength or physical performance and reported no significant differences in miR‐486, miR‐133a, miR‐146a or miR‐21 levels compared to the normal group [21]. Moreover, miRNAs exhibit significant variability in their expression levels across individuals, contributing to increased gene expression variability within human populations. This variability arises from differences in miRNA expression, genetic polymorphisms in miRNA loci and mutations in miRNA target sites [28]. These reasons may account for the discrepancies with our results; therefore, further studies with larger sample sizes are imperative.

In our study, the DE miRNAs, miR‐144‐3p, miR‐142‐3p and miR‐122‐3p overlapped across at least two comparison groups. Notably, miR‐144‐3p showed consistent results in validation analysis, whereas miR‐142‐3p and miR‐122‐3p showed inconsistent results. In previous studies, miR‐144‐3p has been proposed as an important regulator of pro‐inflammatory cytokines in several diseases, such as osteoarthritis, atherosclerosis and cancer [29, 30, 31]; however, its role in skeletal muscle aging remains unclear. A recent study reported that the expression levels of miR‐144‐3p were relatively higher in individuals with sarcopenia than in those without it, although significant differences were not observed [32]. In addition, miR‐144‐3p has been reported to be highly expressed in patients with neurodegenerative diseases, including amyotrophic lateral sclerosis (ALS), which is characterized by motor neuron loss [33]. These findings indicate that the elevated levels of miR‐144‐3p observed in the low muscle strength‐only group may be associated with impaired motor nerve function. KEGG pathway analysis suggested a potential association between miR‐144‐3p and neurotrophin signalling (*p* = 0.0647). Considering that older adults with neurological dysfunctions such as hemiplegia, paraplegia or Parkinson's disease were excluded from our study, this association suggests that identifying neuro‐related signalling imbalances at the molecular level at an early stage may facilitate the prevention of muscle strength decline.

miR‐144‐3p was associated with FoxO and insulin signalling pathways in KEGG pathway analysis. Notably, miR‐144‐3p has been reported to negatively regulate FOXO1, a transcription factor involved in autophagy and the ubiquitin–proteasome system, both of which are involved in muscle protein degradation [34]. In parallel, miR‐144‐3p also targets PTEN, a negative regulator of the PI3K/Akt signalling pathway. Inhibition of PTEN by miR‐144‐3p results in the activation of Akt signalling, thereby enhancing insulin‐stimulated anabolic processes [35]. The miR‐144‐3p–mediated suppression of these molecules may exert a coordinated effect to attenuate muscle protein degradation and promote anabolic signalling. Accordingly, upregulation of miR‐144‐3p in the low muscle strength group may reflect a compensatory response to attenuate FOXO1‐ or PTEN‐mediated catabolic activity, potentially contributing to the preservation of muscle mass despite earlier declines in muscle strength. In contrast, miR‐144 directly inhibits the IRS1, thereby impairing the insulin signalling pathway. miR‐144 has been shown to exhibit high expression levels in the blood samples of individuals with impaired fasting glucose and type 2 diabetes, with a linear increase in expression corresponding to worsening glycaemic status [36]. This may represent the impaired glucose tolerance. Accordingly, the miR‐144 expression levels in the low muscle strength group may be associated with dysfunction in glucose metabolism.

We conducted additional RT‐PCR analyses of candidate miRNAs for sarcopenia identified in our previous systematic review [26]. Among these, miR‐210‐3p was significantly elevated exclusively in the Low MS‐only group. With skeletal muscle aging, a decrease in oxygen demand, along with a reduction in cellular oxygen supply, progressively contributes to the development of hypoxia within muscle tissue [37]. The miR‐210, particularly miR‐210‐3p, exhibits high expression levels under hypoxic conditions and is directly regulated by hypoxia‐inducible factors [38]. Prolonged exposure to hypoxic conditions reduces mitochondrial content in muscle fibres and induces reactive oxygen species formation in skeletal muscle mitochondria [39]. Indeed, previous studies have demonstrated that miR‐210‐3p, which is overexpressed under hypoxic conditions, negatively regulates mitochondrial function. Ismaeel et al. reported that miR‐210 expression in skeletal muscle tissue was inversely correlated not only with mitochondrial respiration rates but also with 6‐min walk distance [40]. Given the previous studies, our findings suggest that the Low MS‐only group may be in a hypoxic state compared to the Low MM‐only and Low PP‐only groups. Thus, miR‐220‐3p may be effective for identifying groups with impaired function and hypoxic conditions.

Our study has a significant strength in being the first to report miRNA profiling for each component of sarcopenia. In particular, a novel biomarker capable of detecting low muscle strength was identified, which is considered clinically significant. Nevertheless, our study had some limitations that need to be recognized. First, we were unable to investigate the detailed medication use of the study participants. To address this, older adults with diseases that could affect small ncRNA analysis were excluded by referring to prior studies [21, 24, 25]. However, despite applying strict exclusion criteria, other unmeasured factors may still have influenced small ncRNA expression. Second, our findings from miRNA sequencing were validated using RT‐PCR only within the same population, which limited our ability to perform cross‐validation with other populations. Third, some participants were excluded from the RT‐PCR validation analyses due to insufficient plasma samples, resulting in a dropout rate of approximately 20%, which may have limited the robustness of the validation results. Fourth, the AUC values were calculated using the same population from whom miRNAs were identified through RNA sequencing and RT‐PCR, which may have contributed to the relatively high AUCs observed in this study. Further studies are warranted to provide external validation of these findings in independent cohorts. Lastly, miRNA profiling was performed using human plasma samples, which presented limitations for accurately identifying the underlying pathways. Experimental validation is required to elucidate the biological functions of the identified miRNAs and to clarify their mechanistic roles in sarcopenia or its components.

## 5 Conclusion

In conclusion, our study examined miRNA profiles in relation to individual components of sarcopenia, specifically muscle mass, muscle strength and physical performance, in older adults. We discovered a novel miRNA, miR‐144‐3p, as an independent biomarker of low muscle strength. Additionally, miR‐210‐3p, previously detected as a candidate miRNA in our systematic review, was confirmed to be exclusively associated with low muscle strength. The identification of muscle strength‐specific miRNAs may serve as potential biomarkers for the early detection of older adults with low muscle strength, thereby facilitating timely interventions aimed at preventing or delaying further declines in muscle strength. It is imperative to examine further longitudinal studies to assess whether the miRNAs identified in our study can serve as predictive biomarkers for muscle strength decline.

## Conflicts of Interest

The authors declare no conflicts of interest.

## Supporting information


**Table S1** List of average miRNA RPM from plasma of LowMM‐only and LowMS‐only groups. Statistically significant fold‐change (BH adjusted *p*‐value < 0.05) are shown **in bold**.
**Table S2.** List of average miRNA RPM from plasma of LowMM‐only and LowPP‐only groups. Statistically significant fold‐change (BH adjusted *p*‐value < 0.05) are shown **in bold**.
**Table S3.** List of average miRNA RPM from plasma of LowMS‐only and LowPP‐only groups. Statistically significant fold‐change (BH adjusted *p*‐value < 0.05) are shown **in bold**.

## Data Availability

The datasets used in this study are not publicly available due to privacy and ethical considerations. Academic inquiries regarding data access should be directed to M.K.
