# Supplementary material for: Circulating miR‐144‐3p as a Novel Independent Biomarker Associated With Low Muscle Strength Among Older Adults
Source: J Cachexia Sarcopenia Muscle. 2025 Jul 29;16(4):e70026. doi: 10.1002/jcsm.70026 (PMC12304732; doi:10.1002/jcsm.70026)
Supplement: Supplementary file 1 — Table S1 List of average miRNA RPM from plasma of LowMM‐only and LowMS‐only groups. Statistically significant fold‐change (BH adjusted p‐value < 0.05) are shown in bold. Table S2. List of average miRNA RPM from plasma of LowMM‐only and LowPP‐only groups. Statistically significant fold‐change (BH adjusted p‐value < 0.05) are shown in bold. Table S3. List of average miRNA RPM from plasma of LowMS‐only and LowPP‐only groups. Statistically significant fold‐change (BH adjusted p‐value < 0.05) are shown in bold. [file JCSM-16-e70026-s001.docx]

**Table S1.**

List of average miRNA RPM from plasma of LowMM-only and LowMS-only groups.

Statistically significant fold-change (BH adjusted p-value< 0.05) are shown ***in bold***.

| **miRNA** | **Low MS-only RPM** | **Low MM-only RPM** | **FC** | **p-value** |
| --- | --- | --- | --- | --- |
| hsa-let-7i-3p | 228.852 | 63.955 | 2.352 | 0.132 |
| **hsa-miR-101-3p** | **484.857** | **131.777** | **3.859** | **0.001** |
| hsa-miR-101-5p | 7.540 | 2.329 | 2.360 | 0.262 |
| hsa-miR-106a-3p | 4.725 | 1.793 | 2.565 | 0.298 |
| **hsa-miR-10b-3p** | **5.888** | **1.417** | **4.785** | **0.035** |
| **hsa-miR-1246** | **3598.653** | **829.506** | **4.453** | **0.014** |
| hsa-miR-125b-5p | 123.501 | 51.503 | 2.639 | 0.065 |
| **hsa-miR-127-3p** | **224.784** | **26.071** | **4.250** | **0.018** |
| hsa-miR-1273c | 3.029 | 6.305 | -2.310 | 0.297 |
| **hsa-miR-1277-3p** | **21.412** | **3.390** | **5.946** | **0.018** |
| hsa-miR-1290 | 509.292 | 246.752 | 2.068 | 0.188 |
| **hsa-miR-1307-5p** | **69.775** | **3.420** | **11.127** | **0.013** |
| hsa-miR-133a-3p | 93.167 | 14.566 | 2.954 | 0.071 |
| hsa-miR-141-3p | 4.754 | 1.992 | 2.483 | 0.216 |
| **hsa-miR-142-3p** | **1741.115** | **484.325** | **3.789** | **0.002** |
| hsa-miR-143-5p | 11.763 | 2.830 | 2.586 | 0.349 |
| **hsa-miR-144-3p** | **667.871** | **64.038** | **10.870** | **0.000** |
| **hsa-miR-144-5p** | **3382.587** | **798.116** | **4.433** | **0.014** |
| hsa-miR-148a-3p | 702.481 | 222.432 | 2.286 | 0.121 |
| hsa-miR-15a-3p | 20.201 | 9.754 | 2.015 | 0.298 |
| **hsa-miR-17-3p** | **41.845** | **8.803** | **3.186** | **0.014** |
| hsa-miR-18b-5p | 4.499 | 1.651 | 2.346 | 0.298 |
| hsa-miR-190a-5p | 79.695 | 36.406 | 2.366 | 0.102 |
| hsa-miR-205-5p | 8.060 | 2.995 | 3.354 | 0.310 |
| hsa-miR-2355-5p | 19.912 | 9.747 | 2.087 | 0.291 |
| **hsa-miR-301a-3p** | **143.961** | **54.036** | **2.852** | **0.035** |
| hsa-miR-301b-3p | 11.643 | 4.165 | 2.398 | 0.148 |
| hsa-miR-30a-5p | 19.369 | 7.787 | 2.705 | 0.061 |
| hsa-miR-338-3p | 29.539 | 7.870 | 2.408 | 0.102 |
| hsa-miR-3611 | 4.035 | 1.522 | 2.527 | 0.328 |
| hsa-miR-3613-5p | 354.520 | 176.791 | 2.132 | 0.328 |
| hsa-miR-3617-3p | 1.974 | 4.924 | -2.211 | 0.262 |
| hsa-miR-370-3p | 57.996 | 13.178 | 2.260 | 0.328 |
| **hsa-miR-378a-3p** | **308.319** | **68.151** | **2.744** | **0.014** |
| hsa-miR-378c | 44.669 | 9.843 | 2.488 | 0.121 |
| hsa-miR-378i | 10.710 | 1.570 | 2.814 | 0.188 |
| hsa-miR-4271 | 16.063 | 3.553 | 2.721 | 0.259 |
| hsa-miR-4306 | 9.938 | 4.607 | 2.271 | 0.262 |
| hsa-miR-4449 | 59.705 | 11.084 | 3.275 | 0.069 |
| hsa-miR-4485-5p | 33.594 | 6.119 | 3.120 | 0.188 |
| hsa-miR-4644 | 6.802 | 1.114 | 2.793 | 0.328 |
| hsa-miR-4738-3p | 0.954 | 2.319 | -2.233 | 0.298 |
| **hsa-miR-491-5p** | **77.282** | **8.005** | **4.776** | **0.029** |
| **hsa-miR-545-5p** | **17.223** | **5.702** | **2.761** | **0.050** |
| hsa-miR-548bc | 3.153 | 1.226 | 2.213 | 0.298 |
| hsa-miR-548w | 2.722 | 1.048 | 2.175 | 0.339 |
| hsa-miR-590-5p | 111.461 | 49.915 | 2.245 | 0.095 |
| hsa-miR-627-5p | 110.978 | 35.574 | 3.123 | 0.065 |
| **hsa-miR-660-5p** | **779.797** | **310.266** | **2.579** | **0.021** |
| hsa-miR-6734-3p | 9.782 | 2.382 | 2.337 | 0.333 |
| hsa-miR-874-3p | 143.074 | 43.175 | 2.001 | 0.142 |
| **hsa-miR-99a-5p** | **15.223** | **5.366** | **2.836** | **0.025** |
| *Abbreviations*: low muscle mass; Low MM, low muscle strength; Low MS, fold change; FC, Reads per million mapped reads; RPM. | | | | |

**Table S2.**

List of average miRNA RPM from plasma of LowMM-only and LowPP-only groups.

Statistically significant fold-change (BH adjusted p-value< 0.05) are shown ***in bold***.

| **miRNA** | **Low PP-only RPM** | **Low MM-only RPM** | **FC** | **p-value** |
| --- | --- | --- | --- | --- |
| hsa-miR-101-3p | 364.009 | 131.777 | 2.210 | 0.105 |
| hsa-miR-106a-3p | 6.929 | 1.793 | 2.356 | 0.414 |
| hsa-miR-10b-3p | 6.871 | 1.417 | 3.003 | 0.132 |
| **hsa-miR-122-3p** | **105.958** | **15.160** | **5.788** | **0.035** |
| hsa-miR-1277-3p | 14.756 | 3.390 | 2.569 | 0.414 |
| hsa-miR-1287-5p | 5.527 | 2.477 | 2.322 | 0.342 |
| hsa-miR-1290 | 711.842 | 246.752 | 2.204 | 0.199 |
| **hsa-miR-142-3p** | **1470.752** | **484.325** | **2.646** | **0.035** |
| **hsa-miR-144-3p** | **248.815** | **64.038** | **3.152** | **0.035** |
| hsa-miR-15a-3p | 23.459 | 9.754 | 2.063 | 0.301 |
| hsa-miR-188-5p | 11.779 | 5.225 | 2.130 | 0.365 |
| hsa-miR-3200-3p | 10.720 | 3.544 | 2.807 | 0.105 |
| **hsa-miR-338-3p** | **34.330** | **7.870** | **3.242** | **0.045** |
| **hsa-miR-33a-5p** | **46.792** | **14.879** | **2.748** | **0.045** |
| hsa-miR-33b-5p | 19.566 | 8.171 | 2.358 | 0.386 |
| hsa-miR-3611 | 4.669 | 1.522 | 2.222 | 0.432 |
| hsa-miR-378i | 4.388 | 1.570 | 2.400 | 0.369 |
| hsa-miR-4484 | 1.746 | 3.103 | -2.536 | 0.414 |
| hsa-miR-6126 | 16.880 | 27.511 | -2.061 | 0.314 |
| hsa-miR-660-5p | 878.051 | 310.266 | 2.233 | 0.105 |
| hsa-miR-6780b-5p | 1.087 | 2.246 | -2.006 | 0.432 |
| *Abbreviations*: low muscle mass; Low MM, low physical performance; Low PP, fold change; FC, Reads per million mapped reads; RPM. | | | | |

**Table S3.**

List of average miRNA RPM from plasma of LowMS-only and LowPP-only groups.

Statistically significant fold-change (BH adjusted p-value< 0.05) are shown ***in bold***.

| **miRNA** | **Low PP-only RPM** | **Low MS-only RPM** | **FC** | **p-value** |
| --- | --- | --- | --- | --- |
| **hsa-miR-122-3p** | **105.958** | **12.568** | **7.153** | **0.018** |
| hsa-miR-1246 | 1904.584 | 3598.653 | -2.431 | 0.741 |
| hsa-miR-127-3p | 27.282 | 224.784 | -3.994 | 0.102 |
| hsa-miR-1287-5p | 5.527 | 3.020 | 2.095 | 0.741 |
| hsa-miR-1307-3p | 219.429 | 970.712 | -2.341 | 0.410 |
| hsa-miR-1307-5p | 4.575 | 69.775 | -6.961 | 0.102 |
| hsa-miR-133a-3p | 19.654 | 93.167 | -2.601 | 0.529 |
| hsa-miR-133b | 7.714 | 30.168 | -2.120 | 0.741 |
| hsa-miR-144-3p | 248.815 | 667.871 | -3.769 | 0.259 |
| hsa-miR-144-5p | 1627.734 | 3382.587 | -2.869 | 0.472 |
| hsa-miR-148a-3p | 262.392 | 702.481 | -2.282 | 0.307 |
| hsa-miR-190a-5p | 47.247 | 79.695 | -2.267 | 0.562 |
| hsa-miR-301a-3p | 90.377 | 143.961 | -2.071 | 0.741 |
| hsa-miR-412-3p | 2.028 | 0.478 | 2.361 | 0.410 |
| hsa-miR-4485-5p | 4.602 | 33.594 | -4.884 | 0.278 |
| hsa-miR-4738-3p | 2.002 | 0.954 | 2.033 | 0.931 |
| hsa-miR-491-5p | 8.947 | 77.282 | -3.565 | 0.278 |
| hsa-miR-502-5p | 2.208 | 0.909 | 2.234 | 0.741 |
| hsa-miR-545-3p | 2.217 | 5.189 | -2.464 | 0.741 |
| hsa-miR-545-5p | 11.074 | 17.223 | -2.073 | 0.838 |
| hsa-miR-576-3p | 3.988 | 7.318 | -2.161 | 0.741 |
| *Abbreviations*: low muscle strength; Low MS, low physical performance; Low PP, fold change; FC, Reads per million mapped reads; RPM. | | | | |

**Additional references**

S1. Won CW, Lee S, Kim J, et al. Korean frailty and aging cohort study (KFACS): cohort profile. BMJ Open. Apr 22 2020;10(4):e035573. doi:10.1136/bmjopen-2019-035573

S2. Kim M, Kim H. Accuracy of segmental multi-frequency bioelectrical impedance analysis for assessing whole-body and appendicular fat mass and lean soft tissue mass in frail women aged 75 years and older. Eur J Clin Nutr. Apr 2013;67(4):395-400. doi:10.1038/ejcn.2013.9

S3. Kim M, Shinkai S, Murayama H, Mori S. Comparison of segmental multifrequency bioelectrical impedance analysis with dual-energy X-ray absorptiometry for the assessment of body composition in a community-dwelling older population. Geriatr Gerontol Int. Aug 2015;15(8):1013-22. doi:10.1111/ggi.12384

S4. Wingo BC, Barry VG, Ellis AC, Gower BA. Comparison of segmental body composition estimated by bioelectrical impedance analysis and dual-energy X-ray absorptiometry. Clinical nutrition ESPEN. Dec 2018;28:141-147. doi:10.1016/j.clnesp.2018.08.013

S5. Narasimhan A, Ghosh S, Stretch C, et al. Small RNAome profiling from human skeletal muscle: novel miRNAs and their targets associated with cancer cachexia. J Cachexia Sarcopenia Muscle. Jun 2017;8(3):405-416. doi:10.1002/jcsm.12168

S6. Kim M, Won CW. Sarcopenia in Korean Community-Dwelling Adults Aged 70 Years and Older: Application of Screening and Diagnostic Tools From the Asian Working Group for Sarcopenia 2019 Update. J Am Med Dir Assoc. Jun 2020;21(6):752-758. doi:10.1016/j.jamda.2020.03.018

S7. Kim M, Won CW, Kim M. Muscular grip strength normative values for a Korean population from the Korea National Health and Nutrition Examination Survey, 2014-2015. PLoS One. 2018;13(8):e0201275. doi:10.1371/journal.pone.0201275

S8. Gatt I, Smith-Moore S, Steggles C, Loosemore M. The Takei Handheld Dynamometer: An Effective Clinical Outcome Measure Tool for Hand and Wrist Function in Boxing. Hand (N Y). May 2018;13(3):319-324. doi:10.1177/1558944717707831

S9. Kim M, Won CW. Combinations of gait speed testing protocols (automatic vs manual timer, dynamic vs static start) can significantly influence the prevalence of slowness: Results from the Korean Frailty and Aging Cohort Study. Archives of gerontology and geriatrics. Mar-Apr 2019;81:215-221. doi:10.1016/j.archger.2018.12.009

S10. Cruz-Jentoft AJ, Bahat G, Bauer J, et al. Sarcopenia: revised European consensus on definition and diagnosis. Age Ageing. Jan 1 2019;48(1):16-31. doi:10.1093/ageing/afy169

S11. Dufourd T, Robil N, Mallet D, et al. Plasma or serum? A qualitative study on rodents and humans using high-throughput microRNA sequencing for circulating biomarkers. Biol Methods Protoc. 2019;4(1):bpz006. doi:10.1093/biomethods/bpz006

S12. Dalman MR, Deeter A, Nimishakavi G, Duan ZH. Fold change and p-value cutoffs significantly alter microarray interpretations. BMC Bioinformatics. Mar 13 2012;13 Suppl 2(Suppl 2):S11. doi:10.1186/1471-2105-13-s2-s11

S13. Bryant RJ, Pawlowski T, Catto JW, et al. Changes in circulating microRNA levels associated with prostate cancer. Br J Cancer. Feb 14 2012;106(4):768-74. doi:10.1038/bjc.2011.595

S14. Liu HW, Cheng HC, Tsai SH, Sun WH. Effect of Progressive Resistance Training on Circulating Adipogenesis-, Myogenesis-, and Inflammation-Related microRNAs in Healthy Older Adults: An Exploratory Study. Gerontology. 2020;66(6):562-570. doi:10.1159/000510148

S15. Liu R, Chen X, Du Y, et al. Serum microRNA expression profile as a biomarker in the diagnosis and prognosis of pancreatic cancer. Clin Chem. Mar 2012;58(3):610-8. doi:10.1373/clinchem.2011.172767
